# The Mediating Role of Appetitive Traits in the Relationship Between Psychological Distress and Body Mass Index of Malaysian Adults

**DOI:** 10.1002/brb3.71327

**Published:** 2026-04-06

**Authors:** Seok Tyug Tan

**Affiliations:** ^1^ Jeffrey Cheah School of Medicine and Health Sciences Monash University Malaysia Bandar Sunway Selangor Malaysia; ^2^ Faculty of Health and Life Sciences Management and Science University Shah Alam Selangor Malaysia

**Keywords:** appetitive traits, body mass index, mediation analysis, psychological distress

## Abstract

**Objective:**

This study investigates the mediating role of appetitive traits in the relationship between psychological distress and Body Mass Index (BMI) of Malaysian adults.

**Methodology:**

A cross‐sectional study involving adults aged 18 to 64 years was conducted, whereby respondents were recruited through convenience sampling in the Klang Valley, Malaysia. Respondents were required to report their socio‐demographics, including sex, age, ethnicity, marital status, highest educational attainment, and monthly income in Malaysian Ringgit (RM). Psychological distress was evaluated using the Depression, Anxiety, and Stress Scale 21 (DASS‐21), while the appetitive traits were assessed using the 35‐item Adults Eating Behavior Questionnaire (AEBQ). Height (cm) was measured using the portable SECA 213 stadiometer, while body weight (kg) was quantified using the HN‐289 Omron digital body weight scale. All measurements were recorded to the nearest 0.1 unit, and BMI was calculated using the measured body height and weight.

**Results:**

Three independent path analyses were conducted to examine the mediating role of appetitive traits in the relationship between psychological distress and BMI. This study revealed that the positive correlation between psychological distress and BMI was partially mediated by emotional overeating. Moreover, psychological distress was also found to be positively and independently correlated with BMI.

**Conclusion:**

To prevent individuals with psychological distress from engaging in emotional overeating and subsequently increasing BMI, interventions should focus on providing effective strategies. This may include promoting physical activity and offering emotional regulation workshops to manage negative emotions. Nutrition education and counseling can also be beneficial for individuals who experience emotional eating in developing healthier dietary practices.

## Graphical abstract

**The path** **analysis of the correlations between psychological distress, emotional overeating, and Body Mass Index (BMI)**.

Main findings

• The positive correlation between psychological distress and body mass index was partially mediated by emotional overeating.

• Psychological distress was also positively and independently correlated with BMI.

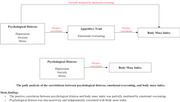

## 1 Introduction

Literature has consistently reported that the unprecedented COVID‐19 pandemic has negatively affected psychological well‐being, eating‐related behavioral patterns, and body weight status (Kannan and Tan 2024; McLean et al. 2022; Willig et al. 2025). Regrettably, these negative impacts seem to have persisted beyond the endemic phase. For instance, the national prevalence of depression in Malaysia has experienced a massive increase due to the COVID‐19 pandemic, rising from 2.3% among adults aged 18 years and above before the pandemic (Institute for Public Health 2020) to the current prevalence of 4.6% among adults aged 16 years and above (Institute for Public Health 2024). A similar trend is also observed for eating‐related behavioral patterns, with young adults in Malaysia exhibiting higher levels of cognitive restraint, uncontrolled eating, and emotional eating during the transition to the endemic phase compared to pre‐pandemic levels (Tan et al. 2024). At the same time, the proportion of overweight or obese adults also saw a sharp increase during the same period, rising from 50.1% before the pandemic (Institute for Public Health 2020) to the current proportion of 54.4% (Institute for Public Health 2024).

Although pandemic‐related changes in eating‐related behavioral patterns have been well‐documented, there is a lack of studies examining appetitive traits among Malaysian adults. Appetitive traits are defined as a set of stable and genetically influenced predispositions toward food approach and avoidance (Hunot et al., 2016). However, it is important to note that individuals with these traits do not inevitably overconsume or gain weight, as behavioral factors (such as physical activity levels and dietary patterns), environmental factors (such as access to energy‐dense foods), and psychological factors (such as psychological distress) also play a critical role in shaping body weight (Schrempft et al. 2025). To the best of the author's knowledge, the Adults Eating Behavior Questionnaire (AEBQ) is among the most commonly used instruments for assessing appetitive traits in adults. In general, it measures two dimensions of appetitive traits: food approach traits (including hunger, food responsiveness, emotional overeating, and enjoyment of food) and food avoidance traits (including satiety responsiveness, emotional undereating, food fussiness, and slowness in eating) (Hunot et al. 2016; Mallan et al. 2017).

While previous studies have examined the correlation between psychological distress and body weight status, and between appetitive traits and body weight status, no study has simultaneously explored the relationships among these three variables. Overall, emerging evidence suggests a bidirectional relationship between psychological distress and body weight status. For instance, Wang et al. (2023) demonstrated that adults who are persistently obese throughout adulthood have a higher risk of depression.Conversely, psychological distress contributes to weight gain through the engagement in maladaptive eating behavior as a coping mechanism (particularly emotional eating), triggering hormonal changes related to appetite and reducing physical activity (Arjmand et al. 2023; Hill et al. 2022; Steptoe and Frank 2023).

The relationship between appetitive traits and body weight status appears to vary by trait dimension. In Australian adults, higher scores in food avoidance traits (satiety responsiveness, emotional undereating, and slowness in eating) were associated with lower body mass index (BMI). In contrast, higher scores in emotional overeating (a food‐approach trait) were associated with higher BMI (Mallan et al. 2017). Even though no existing studies have examined the mediating role of appetitive traits in the relationship between psychological distress and BMI, emerging evidence from the literature on maladaptive eating behaviors as mediators between psychological distress and body weight suggests that such mediation is plausible. Therefore, this study investigates the mediating role of appetitive traits in the relationship between psychological distress and BMI among Malaysian adults.

## 2 Methodology

### 2.1 Study Design and Population

A cross‐sectional study was conducted in the Klang Valley of Malaysia. The Klang Valley (Kuala Lumpur, Selangor, and Putrajaya) was selected as the study location because these areas have the highest adult populations among all states in Malaysia (Department of Statistics Malaysia 2023). Adults aged 18–64 years residing in the Klang Valley, free from clinically diagnosed mental and eating disorders, and physically healthy were recruited for this study using convenience sampling from housing areas, workplaces, shopping malls, and markets.

The required sample size was quantified using G*Power software (version 3.1) with the application of a medium effect size (*f* ^2^ = 0.15) (Cohen 1988), a significance level (α) of 0.05, and a desired statistical power of 0.95. After accounting for a 20% dropout rate from 184 respondents, as suggested by the software, the study needed to recruit at least 221 adults residing in the Klang Valley. Informed consent was obtained from the respondents before the commencement of data collection, and ethical approval was granted by the Research Ethics Committee of Management and Science University with the reference number MSU‐RMC‐02/FR01/06/L1/040.

### 2.2 Socio‐Demographics of Adults

Respondents were required to report their sex (male/female), age, ethnicity (Malay/Chinese/Indian/Others), marital status (unmarried/married), highest educational attainment (primary/secondary/tertiary), and monthly income in Malaysian Ringgit (RM). Age and monthly earned income were collected as continuous variables and recategorized for descriptive reporting of sociodemographic characteristics (Table 1).

**TABLE 1:** Socio‐demographic characteristics of adults.

Socio‐demographic characteristic	*n* (%)	Mean ± Standard Deviation
**Sex**		
Male	65 (14.7)	—
Female	378 (85.3)	
**Age (years old)**		
20‐29	166 (37.5)	
30‐39	244 (55.1)	31.11 ± 5.80
40‐49	25 (5.6)	
50‐59	8 (1.8)	
**Ethnicity**		
Malay	248 (56.0)	
Chinese	89 (20.0)	—
Indian	84 (19.0)	
Others (*Bumiputera* of Sabah/Sarawak or mixed race)	22 (5.0)	
**Marital Status**		
Unmarried	129 (29.1)	—
Married	314 (70.9)	
**Educational attainment**		
Primary and secondary	46 (10.4)	—
Tertiary	397 (89.6)	
**Monthly income (RM)** ^1^		
No income	54 (12.2)	
< RM 5000	270 (60.9)	3382.45 ± 2755.77
≥ RM 5000	119 (26.9)	

^1^ 1 USD = RM 3.94 (as of January 2026).

### 2.3 Psychological Distress Among Adults

The psychological distress among adults was examined using the validated Depression, Anxiety, and Stress Scale 21 (DASS‐21) (Henry and Crawford 2005). The instrument consists of 21 items across three subscales: depression (7‐item), anxiety (7‐item), and stress (7‐item). Items are rated on a 4‐point Likert scale ranging from 0 (“did not apply to me at all”) to 3 (“applied to me very much, or most of the time”). The final scores were calculated by summing the scores from each subscale and multiplying by two. These final scores were subsequently categorized into normal, mild, moderate, severe, and extremely severe according to the suggested cut‐off points. The reliability of DASS‐21 in this study was excellent (Cronbach's alpha = 0.959).

### 2.4 Appetitive Traits Among Adults

Appetitive traits were assessed using the 35‐item Adults Eating Behavior Questionnaire (AEBQ) developed by Hunot et al. (2016). Respondents were required to rate all questions on a 5‐point Likert scale, ranging from strongly disagree (1 point) to strongly agree (5 points). Reverse‐scored items were inverted to maintain consistency in scoring. These questions were further subcategorized into eight appetite traits: hunger (5‐item), food responsiveness (4‐item), emotional overeating (5‐item), enjoyment of food (3‐item), satiety responsiveness (4‐item), emotional undereating (5‐item), food fussiness (5‐item), and slowness in eating (4‐item). The mean score of an appetitive trait was computed by summing the scores from all items and dividing by the number of items within that specific appetitive trait. For food approach traits (hunger, food responsiveness, emotional overeating, and enjoyment of food), higher scores indicate greater tendencies of that appetitive trait. In contrast, for food‐avoidant traits (satiety responsiveness, emotional undereating, food fussiness, and slowness in eating), lower scores are typically indicative of more problematic eating behaviors.

### 2.5 Body Mass Index (BMI) of Adults

Height (cm) was measured using the portable SECA 213 stadiometer, while body weight (kg) was quantified using the HN‐289 Omron digital body weight scale. The respondents were asked to empty their pockets and remove extra clothing, accessories, and shoes before the measurements were taken to ensure accuracy. All measurements were recorded to the nearest 0.1 unit. The Body Mass Index (BMI) of the respondents was calculated and further categorized into underweight (< 18.5 kg/m^2^), normal (18.5–22.9 kg/m^2^), overweight (23.0–24.9 kg/m^2^) and obese (≥ 25.0 kg/m^2^) (World Health Organization, 2000).

### 2.6 Data Analysis

Data were analyzed using IBM SPSS Statistics version 29.0 (IBM Corp., Armonk, NY, USA). Frequency, percentage, mean, and standard deviation (collectively known as descriptive statistics) were used to describe the variables as appropriate. In view of the fact that several studies have excluded the Hunger subscale from the AEBQ due to its poor psychometric properties (Hunot et al. 2016; Hunot‐Alexander et al. 2022; Kuno et al. 2024; Mallan et al. 2017), confirmatory factor analysis was conducted to examine the structural validity of the food approach trait of the AEBQ in the Malaysian adult sample. The Hunger subscale can be considered suitable for inclusion in the AEBQ if it demonstrates good internal reliability (Cronbach's alpha ≥ 0.60), corrected item‐total correlations ≥ 0.30, a Kaiser‐Meyer‐Olkin (KMO) measure ≥ 0.60, a significant Bartlett's test of sphericity (*p* < 0.05), and factor loadings ≥ 0.40 (Sigudla and Maritz 2023).

**FIGURE 1:**
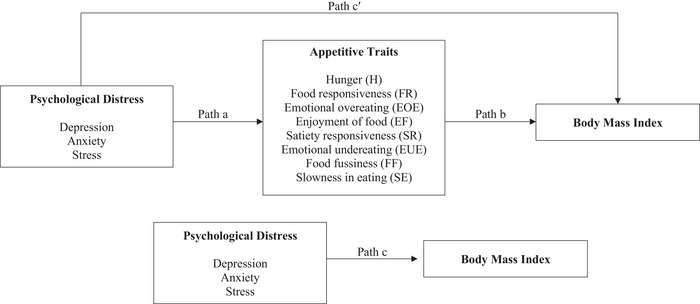
Statistical diagram illustrating the path analyses of the relationships between psychological distress, appetitive traits, and body mass index.

The relationships between psychological distress, appetitive traits, and BMI were assessed using Model 4 of the PROCESS macro for SPSS (Hayes 2022). The eight appetite traits served as mediating variables between psychological distress (independent variable) and BMI (dependent variable) (Figure 1). In this study, three independent path analyses with an adjustment for socio‐demographic variations were conducted: (1) Depression → Appetitive Traits → BMI, (2) Anxiety → Appetitive Traits → BMI, and (3) Stress → Appetitive Traits → BMI. Depression, anxiety, and stress were analyzed as separate predictors rather than combined into a total DASS score because they represent distinct aspects of psychological distress. Conducting three separate path analyses allowed the examination of whether each subscale showed a different pattern of relationship with appetitive traits and BMI. In addition, the findings of Pearson's correlation analysis indicated that depression, anxiety, and stress were highly intercorrelated (*r* = 0.795–0.862, *p* < 0.001); therefore, fitting all three subscales in a single path analysis may introduce multicollinearity. Since linearity assumptions between appetitive traits and BMI were met and multicollinearity among the eight appetitive traits was not a concern (Supplementary Table S1), all traits were entered simultaneously as mediators in each of the three path analyses. ‐

Univariate analyses using either an independent samples *t*‐test (for sex, marital status, and educational attainment), a one‐way ANOVA (for ethnicity), or Pearson's correlation test (for age and monthly earned income) were performed to identify socio‐demographic confounders that could potentially impact the relationships between psychological distress, appetitive traits, and BMI. Socio‐demographic variables that were significant in the univariate analyses (*p* < 0.05) were included as confounders in the path analyses (Supplementary Table S2). Therefore, the three path analyses were carried out with an adjustment for all socio‐demographic variables except for educational attainment. The mediating role of appetitive traits in the relationship between psychological distress and BMI was investigated, with indirect effects assessed through 5000 bootstrap resamples and 95% bias‐corrected confidence intervals. Statistical significance was set at a *p*‐value of less than 0.05 (*p*< 0.05).

## 3 Results

The confirmatory analysis revealed that all items within the food approach traits (including the Hunger subscale) demonstrated good internal consistency (Cronbach's alpha = 0.848). Corrected item‐total correlations ranged from 0.409 to 0.776 (≥ 0.30), indicating that each item contributed adequately to the overall construct. Factor analysis was conducted following a significant Bartlett's test of sphericity (*p* < 0.001) and a KMO measure of 0.778. Exploratory factor analysis using principal axis factoring with direct oblimin rotation showed that all items loaded cleanly onto a single factor, with factor loadings ranging from 0.597 to 0.918 (≥ 0.40). Taken together, these findings indicate that the Hunger subscale exhibits robust psychometric properties and is appropriate for use in the Malaysian context.

Table 1 shows the socio‐demographic characteristics of Malaysian adults who participated in this study. Of the 443 adults, the majority were female (*n* = 378, 85.3%), aged 30–39 years (*n* = 244, 55.1%), of Malay ethnicity (*n* = 248, 56.0%), married (*n* = 314, 70.9%), tertiary educated (*n* = 397, 89.6%), and earned less than RM 5000 per month (*n* = 270, 60.9%).

Table 2 indicates the psychological distress, appetite traits, and body mass index in adults. Of the 443 Malaysian adults, it was observed that approximately three‐fifths (*n* = 257, 58.0%) experienced moderate to extremely severe anxiety (moderate = 28.4%, severe = 12.4%, and extremely severe = 17.2%); slightly more than one‐third (n = 170, 38.4%) presented moderate to extremely severe depression (moderate = 22.6%, severe = 5.9%, and extremely severe = 9.9%); and close to one‐fourth (*n* = 101, 22.8%) suffered moderate to extremely severe stress (moderate = 8.6%, severe = 9.7%, and extremely severe = 4.5%). In relation to appetitive traits, enjoyment of food (EF) (3.78 ± 1.00), food responsiveness (FR) (3.09 ± 0.89), and hunger (H) (2.98 ± 0.80) were the top three traits portrayed by Malaysian adults. In addition, it is also worth mentioning that 59.1% (*n* = 262) of adults in this study were obese.

**TABLE 2:** Psychological distress, appetitive traits, and body mass index in adults.

Variable	*n* (%)	Mean ± Standard Deviation
**Psychological distress**		
**Depression**		
Normal	197 (44.5)	
Mild	76 (17.1)	
Moderate	100 (22.6)	11.79 ± 9.68
Severe	26 (5.9)	
Extremely severe	44 (9.9)	
**Anxiety**		
Normal	142 (32.1)	
Mild	44 (9.9)	
Moderate	126 (28.4)	12.03 ± 8.73
Severe	55 (12.4)	
Extremely severe	76 (17.2)	
**Stress**		
Normal	296 (66.8)	
Mild	46 (10.4)	
Moderate	38 (8.6)	13.90 ± 9.21
Severe	43 (9.7)	
Extremely severe	20 (4.5)	
**Appetite traits**		
Hunger (H)		2.98 ± 0.80
Food responsiveness (FR)		3.09 ± 0.89
Emotional overeating (EOE)		2.72 ± 0.96
Enjoyment of food (EF)	—	3.78 ± 1.00
Satiety responsiveness (SR)		2.67 ± 0.75
Emotional undereating (EUE)		2.86 ± 0.92
Food fussiness (FF)		2.57 ± 0.72
Slowness in eating (SE)		2.82 ± 0.81
**Body Mass Index**		
Underweight	28 (6.3)	
Normal	101 (22.8)	27.06 ± 6.15
Overweight	52 (11.8)	
Obese	262 (59.1)	

Table 3 delineates the path analyses of the relationships between psychological distress, appetitive traits, and body mass index in adults. The findings of this study revealed that psychological distress (depression, anxiety, and stress) was significantly and positively correlated with hunger (depression: B = 0.008, *p* = 0.046; anxiety: B = 0.015, *p* = 0.001; stress: B = 0.012, *p* = 0.003) and emotional overeating (depression: B = 0.013, *p* = 0.009; anxiety: B = 0.019, *p* = 0.001; stress: B = 0.019, *p* =< 0.001). In addition, significant and positive correlations were also observed between anxiety and stress and food responsiveness (anxiety: B = 0.014, *p* = 0.004; stress: B = 0.015, *p* = 0.001). The correlations between appetitive traits and BMI were examined in path b. Among the eight appetite traits, emotional overeating was significantly and positively correlated with BMI (depression: B = 1.640, *p* < 0.001; anxiety: B = 1.643, *p* < 0.001; stress: B = 1.663, *p* < 0.001). In contrast, slowness in eating was significantly and negatively correlated with BMI (depression: B = −0.735, *p* = 0.038; anxiety: B = −0.737, *p* = 0.038; stress: B = −0.739, *p* = 0.039).

**TABLE 3:** The path analyses of the relationships between psychological distress, appetitive traits, and body mass index in adults.

Path^1^	B	SE	t‐value	*p*‐value	LLCI	ULCI
**Path a**						
Depression→ H	0.008	0.004	2.005	**0.046** ^*^	0.001	0.015
Depression→ FR	0.008	0.004	1.791	0.074	−0.001	0.017
Depression→ EOE	0.013	0.005	2.624	**0.009** ^*^	0.003	0.022
Depression→ EF	−0.007	0.005	−1.434	0.152	−0.017	0.003
Depression→ SR	0.005	0.004	1.222	0.223	−0.003	0.012
Depression→ EUE	−0.001	0.005	−0.167	0.868	−0.010	0.008
Depression→ FF	0.006	0.004	1.573	0.116	−0.001	0.013
Depression→ SE	0.003	0.004	0.775	0.439	−0.005	0.011
Anxiety→ H	0.015	0.004	3.405	**0.001** ^*^	0.006	0.023
Anxiety→ FR	0.014	0.050	2.911	**0.004** ^*^	0.005	0.024
Anxiety→ EOE	0.019	0.005	3.513	**0.001** ^*^	0.008	0.029
Anxiety→ EF	0.002	0.006	0.391	0.696	−0.009	0.013
Anxiety→ SR	0.005	0.004	1.151	0.250	−0.003	0.013
Anxiety→ EUE	−0.001	0.005	−0.153	0.879	−0.011	0.009
Anxiety→ FF	0.002	0.004	0.444	0.657	−0.006	0.010
Anxiety→ SE	0.004	0.005	0.924	0.356	−0.005	0.013
Stress→ H	0.012	0.004	2.967	**0.003** ^*^	0.004	0.020
Stress→ FR	0.015	0.005	3.252	**0.001** ^*^	0.006	0.024
Stress→ EOE	0.019	0.005	3.832	**< 0.001** ^*^	0.009	0.029
Stress→ EF	0.002	0.005	0.385	0.700	−0.008	0.013
Stress→ SR	0.006	0.004	1.602	0.110	−0.001	0.014
Stress→ EUE	−0.002	0.005	−0.450	0.653	−0.012	0.007
Stress→ FF	0.002	0.004	0.432	0.666	−0.006	0.010
Stress→ SE	0.006	0.004	1.349	0.178	−0.003	0.014
**Path b** **Depression**						
H→ BMI	−0.564	0.571	−0.988	0.324	−1.687	0.558
FR→ BMI	−0.614	0.551	−1.115	0.266	−1.697	0.469
EOE→ BMI	1.640	0.367	4.473	**< 0.001** ^*^	0.919	2.361
EF→ BMI	0.453	0.431	1.051	0.294	−0.395	1.301
SR→ BMI	−0.738	0.409	−1.806	0.072	−1.541	0.065
EUE→ BMI	0.088	0.344	0.256	0.798	−0.588	0.765
FF→ BMI	−0.509	0.449	−1.132	0.258	−1.392	0.375
SE→ BMI	−0.735	0.353	−2.080	**0.038** ^*^	−1.430	−0.041
**Anxiety**						
H→ BMI	−0.629	0.572	−1.101	0.271	−1.753	0.494
FR→ BMI	−0.569	0.549	−1.037	0.301	−1.648	0.510
EOE→ BMI	1.643	0.366	4.488	**< 0.001** ^*^	0.924	2.363
EF→ BMI	0.378	0.427	0.886	0.376	−0.461	1.218
SR→ BMI	−0.710	0.408	−1.739	0.083	−1.512	0.092
EUE→ BMI	0.111	0.344	0.322	0.748	−0.565	0.786
FF→ BMI	−0.497	0.449	−1.106	0.269	−1.379	0.386
SE→ BMI	−0.737	0.353	−2.087	**0.038** ^*^	−1.431	−0.043
**Stress**						
H→ BMI	−0.524	0.574	−0.913	0.362	−1.652	0.604
FR→ BMI	−0.576	0.555	−1.038	0.300	−1.667	0.515
EOE→ BMI	1.663	0.369	4.508	**< 0.001** ^*^	0.938	2.388
EF→ BMI	0.335	0.430	0.779	0.436	−0.510	1.181
SR→ BMI	−0.733	0.411	−1.784	0.075	−1.540	0.075
EUE→ BMI	0.124	0.346	0.359	0.720	−0.556	0.804
FF→ BMI	−0.485	0.452	−1.073	0.284	−1.372	0.403
SE→ BMI	−0.739	0.356	−2.076	**0.039** ^*^	−1.438	−0.039
**Path c′**						
Depression→ BMI	0.0844	0.0285	2.9645	**0.0032** ^*^	0.0284	0.1403
Anxiety→ BMI Stress→ BMI	0.0977 0.0620	0.0317 0.0303	3.0859 2.0474	**0.0022** ^*^ **0.0412** ^*^	0.0355 0.0025	0.1599 0.1215
**Path c**						
Depression→ BMI	0.0839	0.0285	2.9435	**0.0034** ^*^	0.0279	0.1399
Anxiety→ BMI	0.1045	0.0318	3.2893	**0.0011** ^*^	0.0421	0.1670
Stress→ BMI	0.0698	0.0302	2.3075	**0.0215** ^*^	0.0103	0.1292

^1^ Refer to Figure 1. All analyses were conducted with the adjustment of sex, age, ethnicity, marital status, and monthly income.

* Statistically significant was considered at *p*< 0.05.

Abbreviations: B = unstandardized regression coefficient, SE = standard error, LLCI = lower limit confidence interval, ULCI = upper limit confidence interval.

The direct effect (path c′) and total effect (path c) of psychological distress on BMI were also investigated in this study. The direct and total effects of psychological distress on BMI were examined in this study. For depression, the direct effect on BMI was significant (B = 0.0844, *p* = 0.0032), and the total effect was also significant (B = 0.0839, *p* = 0.0034); however, it is worth highlighting that the indirect effect was very small (B = 0.0005, approximately 0.6% of the total effect). For anxiety, the direct effect on BMI was B = 0.0977, *p* = 0.0022, and the total effect was B = 0.1045, *p* = 0.0011, with an indirect effect of B = 0.0068 (approximately 6.5% of the total effect). For stress, the direct effect was B = 0.0620, *p* = 0.0412; the total effect was B = 0.0698, *p* = 0.0215; and the indirect effect was B = 0.0078 (approximately 11.2% of the total effect). Moreover, the regression models accounted for the following proportions of variance in BMI: depression *R* ^2^ = 0.1815, anxiety *R* ^2^ = 0.1855, and stress *R* ^2^ = 0.1753. In other words, the regression models explained approximately 17.5–18.6% of the variance in BMI. This indicates that psychological distress contributed to BMI differences, although other factors also played a substantial role. Additional analyses were conducted to identify which appetite trait(s) mediated the positive correlation between psychological distress and BMI (Table 4). Among the eight appetitive traits, emotional overeating was reported to partially mediate the relationship between psychological distress and BMI of Malaysian adults (depression: 95% CI = 0.004–0.042; anxiety: 95% CI = 0.011–0.056; stress: 95% CI = 0.011–0.057). Even though the indirect effects were statistically significant, their small magnitude underscored the need for careful interpretation of the mediation relationships.

**TABLE 4:** Summary of the mediation analyses.

Relationship	Indirect effect	95% bias‐corrected bootstrap confidence intervals		Conclusion
		Boot LLCI	Boot ULCI	
Depression→ H→ BMI	−0.004	−0.017	0.005	No mediation
Depression→ FR→ BMI	−0.005	−0.017	0.005	No mediation
Depression→ EOE→ BMI	0.021	0.004	0.042	Partial mediation
Depression→ EF→ BMI	−0.003	−0.013	0.003	No mediation
Depression→ SR→ BMI	−0.003	−0.012	0.003	No mediation
Depression→ EUE→ BMI	−0.001	−0.004	0.003	No mediation
Depression→ FF→ BMI	−0.003	−0.011	0.003	No mediation
Depression→ SE→ BMI	−0.002	−0.012	0.004	No mediation
Anxiety→ H→ BMI	−0.009	−0.030	0.007	No mediation
Anxiety→ FR→ BMI	−0.008	−0.028	0.007	No mediation
Anxiety→ EOE→ BMI	0.031	0.011	0.056	Partial mediation
Anxiety→ EF→ BMI	0.001	−0.005	0.009	No mediation
Anxiety→ SR→ BMI	−0.003	−0.013	0.003	No mediation
Anxiety→ EUE→ BMI	−0.001	−0.004	0.004	No mediation
Anxiety→ FF→ BMI	−0.001	−0.008	0.005	No mediation
Anxiety→ SE→ BMI	−0.003	−0.013	0.004	No mediation
Stress→ H→ BMI	−0.006	−0.023	0.007	No mediation
Stress→ FR→ BMI	−0.009	−0.029	0.008	No mediation
Stress→ EOE→ BMI	0.032	0.011	0.057	Partial mediation
Stress→ EF→ BMI	0.001	−0.005	0.008	No mediation
Stress→ SR→ BMI	−0.005	−0.014	0.002	No mediation
Stress→ EUE→ BMI	−0.001	−0.005	0.004	No mediation
Stress→ FF→ BMI	−0.001	−0.007	0.005	No mediation
Stress→ SE→ BMI	−0.004	−0.016	0.002	No mediation

Abbreviations: Boot LLCI = bootstrap lower limit confidence interval, Boot ULCI = bootstrap upper limit confidence interval.

## 4 Discussion

This study investigated the mediating role of appetitive traits in the relationship between psychological distress and BMI of adults in Malaysia using three independent path analyses. Overall, the findings revealed that food avoidance traits (satiety responsiveness, emotional undereating, food fussiness, and slowness in eating) did not mediate the relationship between psychological distress and BMI. In contrast, emotional overeating was the only food approach trait that partially mediated the positive correlation between psychological distress (depression, anxiety, and stress) and BMI. Moreover, psychological distress was also found to be positively and independently correlated with BMI.

The prevalence of moderate to extremely severe psychological distress among Malaysian young adults was 37.0% (depression), 45.9% (anxiety), and 19.2% (stress) after the third nationwide COVID‐19 pandemic lockdown in 2022 (Tan et al. 2023). However, it was observed that adults in the current study grappled with a higher prevalence of moderate to extremely severe psychological distress (depression = 38.4%, anxiety = 58.0%, stress = 22.8%) compared to young adults in the previously mentioned study. According to the Department of Statistics Malaysia (2024), the consumer price index (CPI) increased by 9.7 points, from 120.1 in February 2020 (before the emergence of the COVID‐19 pandemic) to 130.9 in October 2024. A higher prevalence of moderate to extremely severe psychological distress reported in this study might be attributed to the rising cost of living, as reflected by the CPI.

The recent National Health and Morbidity Survey 2023 (NHMS 2023) revealed that 54.4% of Malaysians have a BMI equal to or greater than 25 kg/m^2^‐ (Institute for Public Health 2024). Coincidentally, the proportion of obese individuals reported in this study (59.1%) was comparable to that in the NHMS 2023. Physical inactivity, spending more time engaged in sedentary behavior such as sitting, watching television, or using electronic devices for extended periods, and living in obesogenic food environments are among the factors contributing to the high prevalence of obesity among Malaysians (Chan et al. 2017; Phulkerd et al. 2022). Interestingly, three of the four food approach traits (enjoyment of food, food responsiveness, and hunger) emerged as the most prominent appetitive traits in Malaysian adults (Table 2). As food‐approach traits are likely to promote increased energy intake and are associated with higher BMI (French et al. 2012; Mallan et al. 2017), it is anticipated that the prevalence of obesity in Malaysia could be further exacerbated in the coming years in the absence of effective interventions.

The current findings suggest that psychological distress (depression, anxiety, and stress) may intensify perceived hunger and contribute to overeating. In addition, anxiety and stress may also trigger higher food responsiveness (Table 3, path a). Overall, these findings align with several previous studies, which demonstrate that psychological distress heightens emotional overeating (consuming an excessive amount of food to alleviate negative emotions rather than in response to physiological hunger) (Celik Erden et al. 2023; Kannan and Tan 2024) and food responsiveness (more responsive to external food cues such as taste, smell, or appearance) (Tryon et al. 2013). To the best of the author's knowledge, the relationship between psychological distress and perceived hunger remains inconclusive to date. While there are studies indicating that psychological distress is associated with hedonic hunger (consuming food for pleasure rather than in response to physiological hunger) (Mason et al. 2020; Yalçın et al. 2023), only one former study by Huh et al. (2015) observed a temporal positive association between stress and perceived hunger.

Emerging evidence suggests that hormonal changes mediate the relationship between psychological distress and alterations in eating behavior. For instance, studies have shown that elevated stress and anxiety promote the release of cortisol, which drives healthy adults to consume energy‐dense, low‐nutrient, and highly palatable foods (Cay et al. 2018; Hill et al. 2022). In addition, ghrelin and leptin, two hormones that work in opposition to regulate satiety, appetite, and energy balance, are also associated with depression (Lis et al. 2024; Naufel et al. 2021). A study by Mills et al. (2019) indicated that individuals with major depressive disorder (MDD) are prone to leptin resistance. Insufficient circulating appetite‐suppressing leptin may contribute to increased food intake and a reduced ability to control eating. Although levels of the hunger hormone ghrelin were not found to be elevated in individuals with MDD compared to those without the disorder, higher ghrelin levels were associated with a reduced cognitive ability to voluntarily restrict food intake.

Of the eight appetite traits analyzed in this study, only emotional overeating and slowness in eating were correlated with BMI (Table 3, path b). Moreover, emotional overeating partially mediated the positive correlation between psychological distress and BMI (Table 4). The negative correlation between slowness in eating and BMI suggests that individuals who eat at a slower pace tend to have a lower BMI (Hurst and Fukuda 2018). This could be attributed to the fact that eating slowly enhances the body's ability to process satiety signals, thereby preventing overeating and subsequently reducing the risk of weight gain (Simon et al. 2022). The findings also indicated that individuals suffering from psychological distress were more likely to engage in emotional eating and had a higher BMI. Similarly, the literature consistently reports that individuals with psychological distress are more likely to consume energy‐dense and highly palatable comfort foods as coping mechanisms to alleviate negative emotions, including depression, anxiety, and stress. Long‐term engagement in such atypical eating behavior may contribute to weight gain (Arjmand et al. 2023; Dakanalis et al. 2023; Konttinen 2020).

Although psychological distress was positively correlated with hunger and food responsiveness, no correlation was observed between hunger and BMI or between food responsiveness and BMI (Table 3, path a and path b). The lack of correlations may be attributed to food availability. Despite psychological distress causing appetite‐related hormonal changes, consuming energy‐dense and highly palatable foods would not occur in individuals who are hungry and responsive to external cues if such foods are unavailable, resulting in no significant change in BMI (Konttinen 2020). Another noteworthy finding is that psychological distress was positively correlated with the BMI of adults in this study (Table 3, path c and c′). This indicates that adults who experienced high levels of psychological distress were more likely to have a high BMI. In addition to emotional eating and appetite‐related hormonal changes, sleep quality also plays a significant role in explaining the positive correlation between psychological distress and BMI. Emerging evidence suggests that individuals experiencing physiological distress are prone to sleep deprivation. Sleep deprivation, which stimulates ghrelin and suppresses leptin, may contribute to weight gain over time (Akhlaghi and Kohanmoo 2025).

The findings of this study should be interpreted in light of its limitations. Given the multifaceted relationships among psychological distress, appetitive traits, and BMI, future studies should consider measuring intervening factors such as dietary patterns, energy‐dense food availability, health status, appetite‐related hormonal changes, physical activity levels, and sleep quality in adults. The generalizability of the current findings to the broader Malaysian adult population may be limited because this study employed a cross‐sectional design, used convenience sampling for participant recruitment, and collected data solely in the Klang Valley. Furthermore, reliance on self‐reported measures of psychological distress and appetitive traits may introduce bias, including social desirability and recall errors. It is also observed that the sample was demographically skewed (85.3% female, 89.6% tertiary educated, 70.9% married) and exhibited a high prevalence of moderate‐to‐severe anxiety (58.0%) and obesity (59.1%). These characteristics may have affected the variance and covariance structures of the studied variables, and thus, the findings should be interpreted with caution. Another limitation worth highlighting is that causal relationships cannot be inferred from these findings due to the cross‐sectional study design. Despite the limitations mentioned, this study is the first to investigate the mediating role of appetitive traits in the relationship between psychological distress and BMI in adults in Malaysia.

## 5 Conclusion

This study revealed that emotional overeating mediated the positive correlation between psychological distress and BMI. Moreover, psychological distress was also found to be positively and independently correlated with BMI. To prevent individuals with psychological distress from engaging in emotional overeating and subsequently increasing BMI, interventions should focus on providing effective strategies. This may include promoting physical activity and offering emotional regulation workshops to manage negative emotions. Nutrition education and counseling can also be beneficial for individuals who experience emotional eating in developing healthier dietary practices.

## Author Contributions

The author confirms sole responsibility for study conception and design, data collection, analysis and interpretation of results, and manuscript preparation.

## Funding

The author has nothing to report.

## Ethics Statement

Ethical approval was granted by the Research Ethics Committee of Management and Science University with the reference number MSU‐RMC‐02/FR01/06/L1/040.

## Consent

Written informed consent was obtained from all respondents.

## Conflicts of Interest

The author declares no conflicts of interest.

## Supporting information




**Supplementary Tables**: brb371327‐sup‐0001‐TableS1‐S2.docx

## Data Availability

The datasets used and/or analyzed during the current study are available from the corresponding author upon reasonable request.
