# Supplementary material for: The Mediating Role of Appetitive Traits in the Relationship Between Psychological Distress and Body Mass Index of Malaysian Adults
Source: Brain Behav. 2026 Apr 6;16(4):e71327. doi: 10.1002/brb3.71327 (PMC13053306; doi:10.1002/brb3.71327)
Supplement: Supplementary file 1 — Supplementary Tables: brb371327‐sup‐0001‐TableS1‐S2.docx [file BRB3-16-e71327-s001.docx]

**Supplementary**

**Table 1: Correlation matrix of the mediators (appetitive traits)**

| **Appetitive trait** | **Correlation matrix, *r-value*** | | | | | | | **Tolerance^1^** | **Variance Inflation Factor (VIF)^1^** |
| --- | --- | --- | --- | --- | --- | --- | --- | --- | --- |
|  | FR | EOE | EF | SR | EUE | FF | SE |  |  |
| H | -0.545 | -0.313 | -0.099 | -0.099 | -0.165 | -0.136 | -0.062 | 0.330 | 3.035 |
| FR | - | -0.131 | -0.347 | -0.051 | -0.010 | 0.144 | 0.121 | 0.295 | 3.394 |
| EOE | - | - | -0.125 | -0.050 | 0.402 | -0.043 | -0.072 | 0.556 | 1.799 |
| EF | - | - | - | -0.041 | -0.182 | 0.417 | 0.008 | 0.387 | 2.538 |
| SR | - | - | - | - | -0.231 | -0.139 | -0.302 | 0.723 | 1.382 |
| EUE | - | - | - | - | - | 0.022 | -0.134 | 0.684 | 1.462 |
| FF | - | - | - | - | - | - | 0.139 | 0.655 | 1.527 |
| SE | - | - | - | - | - | - | - | 0.819 | 1.221 |

**Abbreviations:** Hunger (H), Food responsiveness (FR), Emotional overeating (EOE), Enjoyment of food (EF), Satiety responsiveness (SR), Emotional undereating (EUE), Food fussiness (FF) and Slowness in eating (SE).

^1^Multicollinearity is not a concern.

**Table 2: Socio-demographic determinants of psychological distress, appetitive traits, and BMI in adults.**

| **Variables** | **t-value/r-value/F-value (*p*-value)** | | | | | |
| --- | --- | --- | --- | --- | --- | --- |
|  | **Sex^1^** | **Age^2^** | **Ethnicity^3^** | **Marital status^1^** | **Educational attainment^1^** | **Monthly income^2^** |
| **Psychological distress**  Depression  Anxiety  Stress | -2.349 (0.019)  -5.036 (< 0.001)  -5.090 (< 0.001) | -0.166 (< 0.001)  -0.180 (< 0.001)  -0.170 (< 0.001) | 1.058 (0.367)  2.099 (0.100)  1.868 (0.134) | 1.502 (0.134)  0.868 (0.386)  1.175 (0.241) | 0.765 (0.445)  1.119 (0.264)  0.791 (0.429) | -0.236 (< 0.001)  -0.243 (< 0.001)  -0.214 (< 0.001) |
| **Appetite traits**  Hunger  Food responsiveness  Emotional overeating  Enjoyment of food  Satiety responsiveness  Emotional undereating  Food fussiness  Slowness in eating | -3.571 (< 0.001)  -3.022 (0.003)  -2.186 (0.029)  0.959 (0.338)  -3.714 (< 0.001)  0.346 (0.730)  -1.479 (0.140)  -2.713 (0.007) | -0.244 (< 0.001)  -0.205 (< 0.001)  -0.060 (0.211)  0.087 (0.066)  -0.066 (0.164)  -0.148 (0.002)  -0.027 (0.570)  -0.051 (0.286) | 5.394 (0.001)  5.978 (< 0.001)  2.516 (0.058)  1.273 (0.283)  0.321 (0.811)  1.502 (0.213)  5.187 (0.002)  0.442 (0.723) | 0.529 (0.597)  1.821 (0.069)  -1.969 (0.050)  1.895 (0.059)  0.365 (0.715)  1.416 (0.157)  -1.904 (0.058)  -0.273 (0.785) | -0.961 (0.337)  -0.560 (0.576)  -0.276 (0.783)  0.104 (0.917)  1.323 (0.187)  -0.701 (0.483)  1.077 (0.282)  -0.164 (0.870) | -0.094 (0.048)  -0.093 (0.051)  0.023 (0.632)  0.051 (0.283)  -0.083 (0.082)  -0.032 (0.499)  -0.079 (0.095)  -0.080 (0.092) |
| **BMI** | -0.460 (0.646) | 0.172 (< 0.001) | 18.840 (< 0.001) | -6.739 (< 0.001) | 1.432 (0.153) | 0.405 (< 0.001) |

^1^Mean difference was determined using independent samples t-test.

^2^Correlation was determined using Pearson’s correlation test.

^3^Mean difference was determined using one-way ANOVA.

Note: Socio-demographic variables that showed a significant difference (*p*< 0.05) were then included as confounders in the path analyses. Therefore, sex, age, ethnicity, marital status, and monthly income were treated as confounders in all three path analyses.
